# Comparison of a Machine Learning Method and Various Equations for Estimating Low-Density Lipoprotein Cholesterol in Korean Populations

**DOI:** 10.3389/fcvm.2022.824574

**Published:** 2022-02-10

**Authors:** Yu-Jin Kwon, Hyangkyu Lee, Su Jung Baik, Hyuk-Jae Chang, Ji-Won Lee

**Affiliations:** ^1^Department of Family Medicine, Yonsei University College of Medicine, Yongin Severance Hospital, Yongin, South Korea; ^2^Mo-Im Kim Nursing Research Institute, College of Nursing, Yonsei University, Seoul, South Korea; ^3^Healthcare Research Team, Health Promotion Center, Gangnam Severance Hospital, Seoul, South Korea; ^4^Department of Internal Medicine, Division of Cardiology, Severance Cardiovascular Hospital, Yonsei University College of Medicine, Seoul, South Korea; ^5^Department of Family Medicine, Yonsei University College of Medicine, Gangnam Severance Hospital, Seoul, South Korea

**Keywords:** low-density lipoprotein, deep neural network, pooled cohort equation, Korean, cardiovascular disease

## Abstract

**Background:**

LDL-C is the primary target of lipid-lowering therapy and used to classify patients by cardiovascular disease risk. We aimed to develop a deep neural network (DNN) model to estimate LDL-C levels and compare its performance with that of previous LDL-C estimation equations using two large independent datasets of Korean populations.

**Methods:**

The final analysis included participants from two independent population-based cohorts: 129,930 from the Gangnam Severance Health Check-up (GSHC) and 46,470 participants from the Korean Initiatives on Coronary Artery Calcification registry (KOICA). The DNN model was derived from the GSHC dataset and validated in the KOICA dataset. We measured our proposed model's performance according to bias, root mean-square error (RMSE), proportion (P)10–P20, and concordance. P was defined as the percentage of patients whose LDL was within ±10–20% of the measured LDL. We further determined the RMSE scores of each LDL equation according to Pooled cohort equation intervals.

**Results:**

Our DNN method has lower bias and root mean-square error than Friedewald's, Martin's, and NIH equations, showing a high agreement with LDL-C measured by homogenous assay. The DNN method offers more precise LDL estimation in all pooled cohort equation strata.

**Conclusion:**

This method may be particularly helpful for managing a patient's cholesterol levels based on their atherosclerotic cardiovascular disease risk.

## Introduction

Cardiovascular disease (CVD) risk assessment is the first step in managing and preventing major vascular events and all-cause mortality (1). Low-density lipoprotein cholesterol (LDL-C) is a major modifiable cardiovascular risk factor (1). According to the recent American College of Cardiology/American Heart Association (ACC/AHA) and European Society of Cardiology and European Atherosclerosis Society (ESC/EAS) guidelines, LDL-C should be strictly managed for primary and secondary prevention of cardiovascular events (2, 3). To best inform clinical decisions and the use of effective therapies, health care providers require a precise and accurate method to measure LDL-C in the clinical setting. Additionally, providers need a method that guides LDL-C management strategies based on a patient's risk of CVD (3, 4). Many working groups recommend setting individualized targets for LDL-C based on a patient's total CVD risk level to manage CVD (2, 3). In 2013, the ACC/AHA developed pooled cohort equations (PCEs) to predict the 10-year risk for atherosclerotic cardiovascular disease (ASCVD) events and recommended the use of these PCEs in treatment for blood cholesterol (5).

Although tests for measuring LDL, such as beta-quantification (BQ) procedure and Vertical Auto Profile (VAP), have been developed, these techniques are more expensive than the standard lipid panel and inappropriate for routine clinical practice (6, 7). Traditionally, LDL-C is estimated using the Friedewald equation, which applies a fixed ratio of triglycerides (TGs) to very low-density lipoprotein (VLDL) cholesterol (8). However, the Friedewald equation underestimates LDL-C at low levels of LDL-C and with high TGs levels (9). Therefore, a novel method was developed by Martin, wherein LDL-C is expressed as follows: total cholesterol—high-density lipoprotein cholesterol—TGs/(strata-specific median VLDL-C:TGs ratio) (10). Nonetheless, neither the Friedewald nor the Martin methods are well-suited to the setting of severe hypertriglyceridemia (10). Recently, Maureen Sampson et al. developed the following new LDL-C equation for patients with hypertriglyceridemia and/or a low level of LDL-C from the National Institutes of Health (NIH) Clinical Center: TC/0.948 – HDL-C/0.971–[TG/8.56 + (TG × non-HDL-C)/2140 – *TG*^2^/16100]−9.44 (11). This new equation also requires independent verification in multiple datasets, particularly in the Asian population. Machine learning with deep neural network (DNN) models has been highlighted for classification systems to diagnose disease because it can represent highly complex data (12). DNN utilizes multiple processing layers to learn representations of data with multiple levels of abstraction (12, 13). Taken together, the application of the machine learning could be a simple task applied in the modern laboratory that is efficient in terms of technology and cost (14). However, several studies have evaluated machine learning for cardiovascular risk assessment including LDL determination. Although several studies have developed machine learning methods to estimate LDL levels (14–16), they created machine learning models with relatively small sample sizes.

In this study, we aimed to develop a DNN-based LDL-C estimating model (LDL-*C*_*DNN*_) and compare the performance of this DNN model with that of previous formulas for LDL-C calculation using two large independent datasets of Korean populations. Furthermore, we aimed to validate the utility of LDL-*C*_*DNN*_ in the group stratified by estimated CVD risk.

## Methods

### Study Population

This study used the data of two independent population-based cohorts: Gangnam Severance Health Check-up (GSHC) dataset and Korean Initiatives on Coronary Artery Calcification (KOICA) registry. The GSHC dataset consisted of retrospective data obtained from 144,910 participants who visited Gangnam Severance Health Check-up for comprehensive health check-ups from March 2, 2007, to March 12, 2020. After excluding participants with missing data for demographics (*n* = 8,795), lifestyle factors (*n* = 106), and laboratory tests (*n* = 6,079), a total of 129,930 participants were included in this analysis.

The KOICA registry dataset contained retrospective, multicenter, observational cohort data obtained from 56,446 patients who underwent a general health examination at one of six healthcare centers in Korea from December 2012 to August 2016 (17). All participants voluntarily signed an informed consent form before the study, and the institutional review boards (IRB) of each study site approved the study protocols. After excluding the participants with missing data for laboratory tests (*n* = 9,976), a total of 46,470 participants were included in this study.

**Figure 1:**
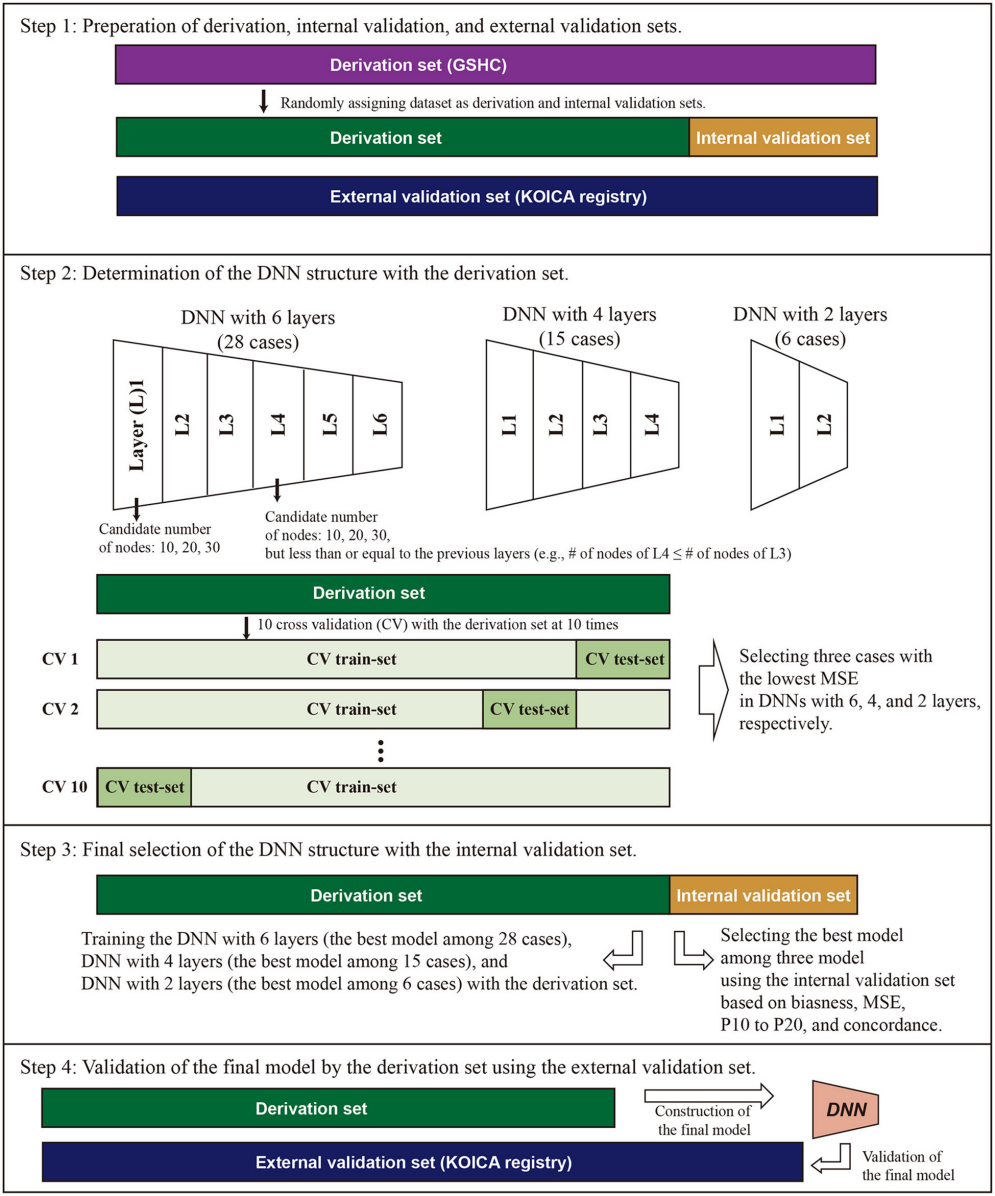
Conceptual schematic for internal and external validation of machine learning models. For internal validation, the Gangnam Severance Health Check-up (GSHC) was reserved for testing the model performance. For external validation, Korean Initiatives on Coronary Artery Calcification (KOICA) registry was used to test the model performance. The DNN consists of six hidden layers, four hidden layers, and two hidden layers, with 30 nodes in each layer. Ten cross-validation was performed to determine the structure of DNN in the derivation set. We selected the best DNN model with lowest mean standard error (MSE) among the three layers. A total of three DNN models were competed. The final model was validated using the external validation set.

This study was conducted in accordance with the Declaration of Helsinki and approved by the Institutional Review Board of Severance Hospital (IRB No. 4-2020-0323). To create a prediction model, 70% of participants of the GSHC were randomly assigned to a derivation dataset. A DNN equation for LDL was developed using the derivation dataset (Figure 1). To validate the model, 30% of GSHC participants were randomly assigned to an internal validation dataset. KOICA registry samples were assigned to an external validation dataset. Data on the history of hypertension, diabetes, and smoking status were obtained from a self-reported questionnaire to both cohort participants.

### Lipid Assessment

All blood samples were collected from the antecubital vein after an overnight fast of at least 8 h. In the GSHC, serum LDL-C was measured by a homogenous direct assay using reagents from Sekisui Medical Corporation (Tokyo, Japan) on a Hitachi 7600 automated analyzer (Hitachi, Tokyo, Japan) until March 17, 2014; after this date, the homogeneous direct assay used reagents from Beckman Coulter Inc. (Brea, CA, USA) on an AU5800 automated analyzer (Beckman Coulter Inc.). In the KOICA registry, data were gathered from three locations: Severance Check-up Healthcare Center, Seoul National University Healthcare System Gangnam Center, and Samsung Medical Center. Serum LDL-C levels were measured by homogenous direct assays using reagents from Sekisui, Beckman, or Roche Diagnostics (Mannheim, Germany) on Hitachi 7600, Modular D2400, or Architect Ci8200 (Abbott, Abbott Park, IL) automated analyzers.

### Assessment of Various LDL Cholesterol Estimation

A number of studies have developed methods to estimate LDL. We adopted three methods: Friedewald (18), the Martin method (10), and NIH methods (11). The Friedewald LDL-C equation was used as TC–HDL-C– (TG/5) in mg/dL. A novel equation for LDL-C was estimated as TC – HDL-C – TG/adjustable factor (based on the non-HDL-C and TG levels derived from a 180-cell 2D table). The NIH equation for LDL-C was estimated as TC/0.948 – HDL-C/0.971 – (TG/8.56 + [TG × non-HDL-C]/2140 – TG^2^/16100)−9.44.

### Determination of DNN Structure and Validation Strategies

Figure 1 describes the overall study scheme that applied in this study. First, we split the GSHC data into a derivation set (70%) and an internal validation set (30%). The KOICA registry used as external validation set. Second, DNN model selection was conducted using the tournament method. We selected the preliminary results as a form of competition among a variety of DNN structures. The candidate DNN structures consisted of six hidden layers, four hidden layers, and two hidden layers. For six hidden layers, the DNN model was set as the pyramid structure. In other words, the candidate number of nodes were 10 nodes for six layers, 20 nodes for four layers, and 30 nodes for two layers, but less than or equal to the previous layers. In the cases of four and two hidden layers, candidates were determined similarly using the above pyramid structure. Ten cross-validation was performed to determine the structure of DNN and to check its performance in the derivation set. Additionally, 90% of the derivation dataset was arranged into a training dataset. Then, a remnant dataset remained as a testing dataset. Notably, the 10 cross-validation analyses were exclusively conducted using only the derivation dataset. We selected the best DNN model with the lowest mean standard error among the three layers (one of six layers, one of four layers, and one of two layer). A total three DNN models were eligible to be entered into the final round.

**Figure 2:**
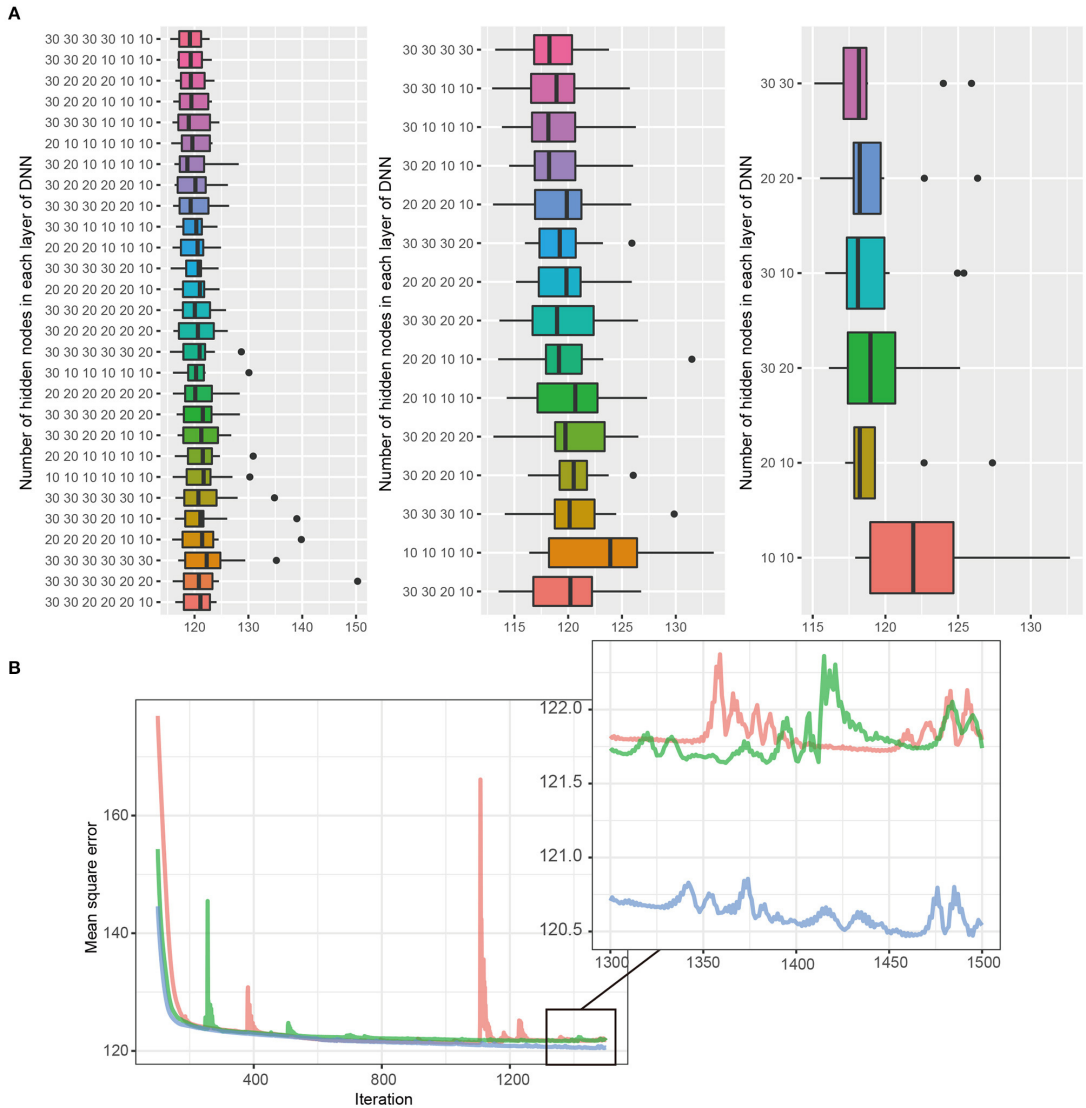
**(A,B)** DNN model selection. The DNN consists of six hidden layers, four hidden layers, and two hidden layers, with 30 nodes in each layer. Black bars, upper margins, and maximum lines in each boxplot indicate the means, one standard deviation (SD), and 1.96 SD deviation values, respectively. The best DNN model among each layer was selected, and the tournament method was used to identify the final model. The 30, 30 in the two-layer model had the lowest MSE and was selected as the final model.

For the third step, these three models (i.e., the best models in layer 6, layer 4, and layer 2) competed, and one model with the best performance was determined to use in this study (Figure 2). Notably, the final round was conducted using the internal validation dataset (30% of GSHC). Finally, we validated the selecting final model using the external validation set according to four accuracy indices.

### Performance Measurement

Our study conducted the cross-validation, a set of methods for measuring the performance of a predictive model on a test dataset. To determine whether the DNN model accurately represents the LDL level, we conducted four model validation methods: bias, RMSE, P10 to P20, and concordance.

Based on previous studies, the bias (estimated LDL – mean value of LDL) of each LDL equation was calculated, and the one-sample *t*-test was used to measure the degree of average bias of each estimation method differing from zero (19, 20). The RMSE is the square root of the mean of the square of all of the error. The most common measure of cross-validation is the RMSE. We also calculated the accuracy using the “P” value. *P* was defined as the percentage of patients whose LDL was within ±10–20% of the measured LDL. We found that P30 was too loose criteria for evaluating clinical accuracy when we reviewed the Hwang et al. study (20). Therefore, we used P10, P15, and P20, and we defined Pn (*n* = 10, 15, and 20) as follows:

$$\begin{array}{r} Pn=\frac{number\;of\;samples\;with\;estimated\;LDL-within\;mean\;LDL\pm n\%}{number\;of\;all\;samples} \end{array}$$

Concordance has been used to test the classification accuracy between estimated LDL and directly measured LDL.

$$\begin{array}{r} Concordance=\frac{\#\;of\;B\cap A}{\#\;of\;A} \end{array}$$

where A are samples with direct measured LDL within a specific range and B are samples with estimated LDL in the same range as directly measured LDL.

To assess the concordance in LDL-C risk classification between estimated and directly measured LDL, we classified LDL-C values into six categories (<99, 100–129, 130–159, 160–189, and ≥ 190 mg/dL) according to previous published dyslipidemia guidelines (21, 22). Because the number of samples below into the <99 mg/dl of LDL category was too small, we integrated them into 100 mg/dl or less. Concordance in classification between estimated LDL and directly measured LDL were tested according to TG classification and non-HDL-C classification with the same method.

### Application of Pooled Cohort Equations

Treatment based on absolute risk determined using combinations of risk factors rather than one value such as LDL-C has been widely accepted since the National Cholesterol Education Program Adult Treatment Panel III guidelines published in 2001. In 2013, the ACC/AHA developed a new risk score, based on major National Heart, Lung, and Blood Institute-funded cohort studies. These PCEs predict 10-year risk of hard ASCVD (23). In 2018, Yadlowsky et al. (24) derived a revised version of 2013 PCEs to improve the clinical accuracy of CVD risk prediction. We used these revised PCEs. The statistical code that we used is available at https://github.com/sanjaybasu/revised-pooled-ascvd. PCE scores were stratified into 20th deciles. Concordance in classification between PCEs and the LDL equation according to 20 categories was examined in the study cohorts.

### Statistical Analysis

Numeric data distribution of overall study population was described as the histogram. Continuous data were presented as the means ± standard deviation and medians (IQR). Categorical data were presented as number (%). General characteristics of three groups (derivation dataset, internal validation dataset, and external validation dataset) were compared using the one-way analysis of variance and Mann-Whitney *U* test for continuous variables. Categorical variables were compared using the chi-square test among the three groups. Additionally, Bonferroni correction was conducted.

To select the combination DNN model for predicting LDL-C, 10-fold cross-validation was performed. Cross-validation is the statistical method to reduce an overfitting problem in the estimating and evaluation of the performance of the models (25). First, the internal data was randomly split into a training dataset (*n* = 38,928) and a test dataset (*n* = 91,002). In 10-fold cross-validation, the internal training set was randomly partitioned into 10 subsets. The cross-validation process was repeated 10 times, with each of 10 subsets used as internal validation data. For selecting the best model, all possible combinations were fitted, and the performance of each model was compared. The model that produced the best prediction performance was selected as the preferred model.

Statistical analysis was conducted using R Statistical Package (Institute for Statistics and Mathematics, Vienna, Austria, version 4.1.0, www.R-project.org). A *p* value <0.05 was used as the significance level.

## Results

**Figure 3:**
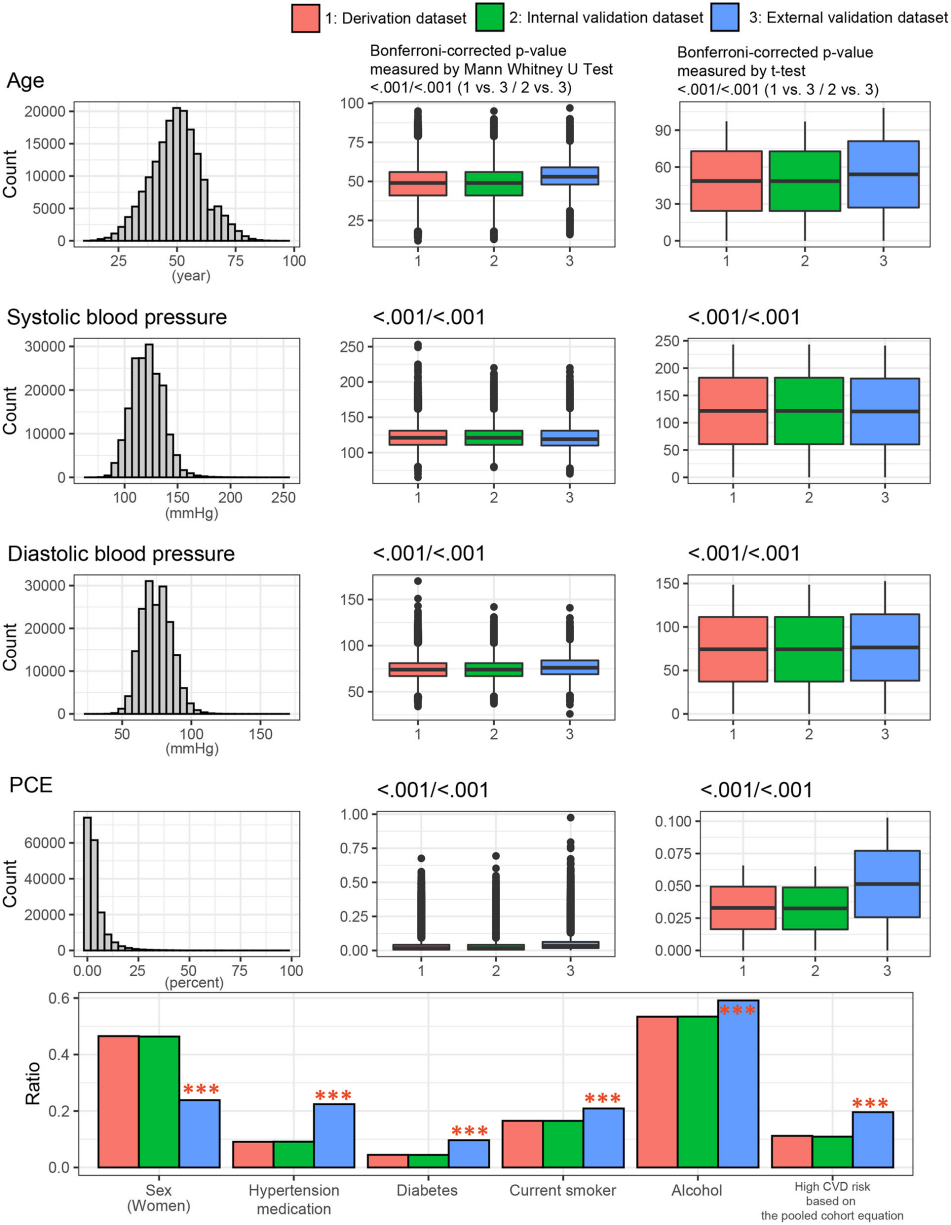
Clinical characteristics of the study population among the three datasets. P values were calculated using the *t*-test and Mann-Whitney *U* test for continuous variables or the chi-square test for categorical variables. ***p < 0.001. PCE, pooled cohort equations.

To identify any differences among the three datasets at baseline, we compared the general characteristics of study participants across the three datasets. Figure 3 presents the general characteristics of the study populations from the three datasets. The distribution of entire dataset was described. The mean age ± standard deviations were 48.6 ± 11.5 years in the derivation dataset, 48.5 ± 11.4 years in the internal dataset, and 54.0 ± 8.9 in the external dataset. The proportions of male participants were 53.5% in the derivation dataset, 53.6% in the internal dataset, and 76.2% in the external dataset. The proportion of high-risk CVD group (PCE ≥20%) were 11.2% in the derivation set, 10.9% in the internal validation set, and 19.6% in the external validation set. Participants in KOICA were more likely to be old, male, have a history of hypertension and diabetes, smoke cigarettes, and have a high CVD risk. No significant differences were found between the derivation and internal validation sets.

**Figure 4:**
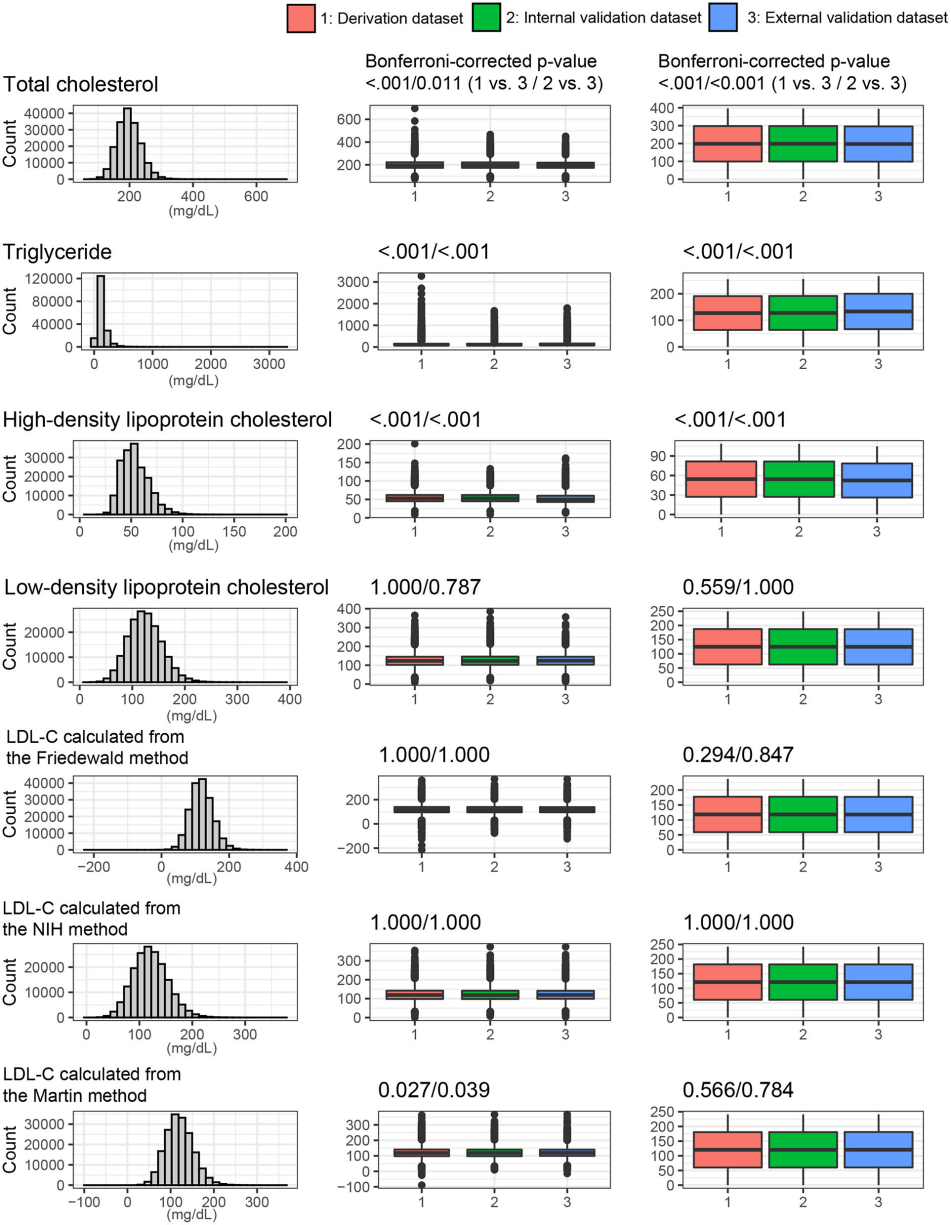
Lipids profiles and LDL values from the various LDL equations. The distribution of whole dataset was described. P values were calculated using the *t*-test and Mann-Whitney *U* test for continuous variables.

Figure 4 shows the lipid profiles of the three datasets. The mean values of total cholesterol were 198.3 ± 37.1 mg/dl in the derivation set, 198.3 ± 37.0 mg/dl in the internal validation set, and 197.1 ± 35.0 mg/dl in the external validation set. The median values (interquartile range [IQR]) of TGs were 105 (75–153) mg/dl in the derivation and internal validation sets and 113 (79–163) mg/dl in the external validation set. The mean values of HDL cholesterol were 54.4 ± 13.2 mg/dl in the derivation set, 54.4 ± 13.1 mg/dl in the internal validation set, and 52.3 ± 13.1 mg/dl in the external validation set. The mean values of directly measured LDL were 124.8 ± 32.4 mg/dl in the derivation set, 124.8 ± 32.5 mg/dl in the internal validation set, and 124.6 ± 31.3 mg/dl in the external validation set. The Korean Initiatives on Coronary Artery Calcification (KOICA) registry exhibited significantly lower levels of total cholesterol and HDL cholesterol but a significantly higher level of TGs. The levels of LDL-C and estimated LDL-C were not significantly different among the three sets. These baseline characteristics are presented in Supplementary Table S1.

**Figure 5:**
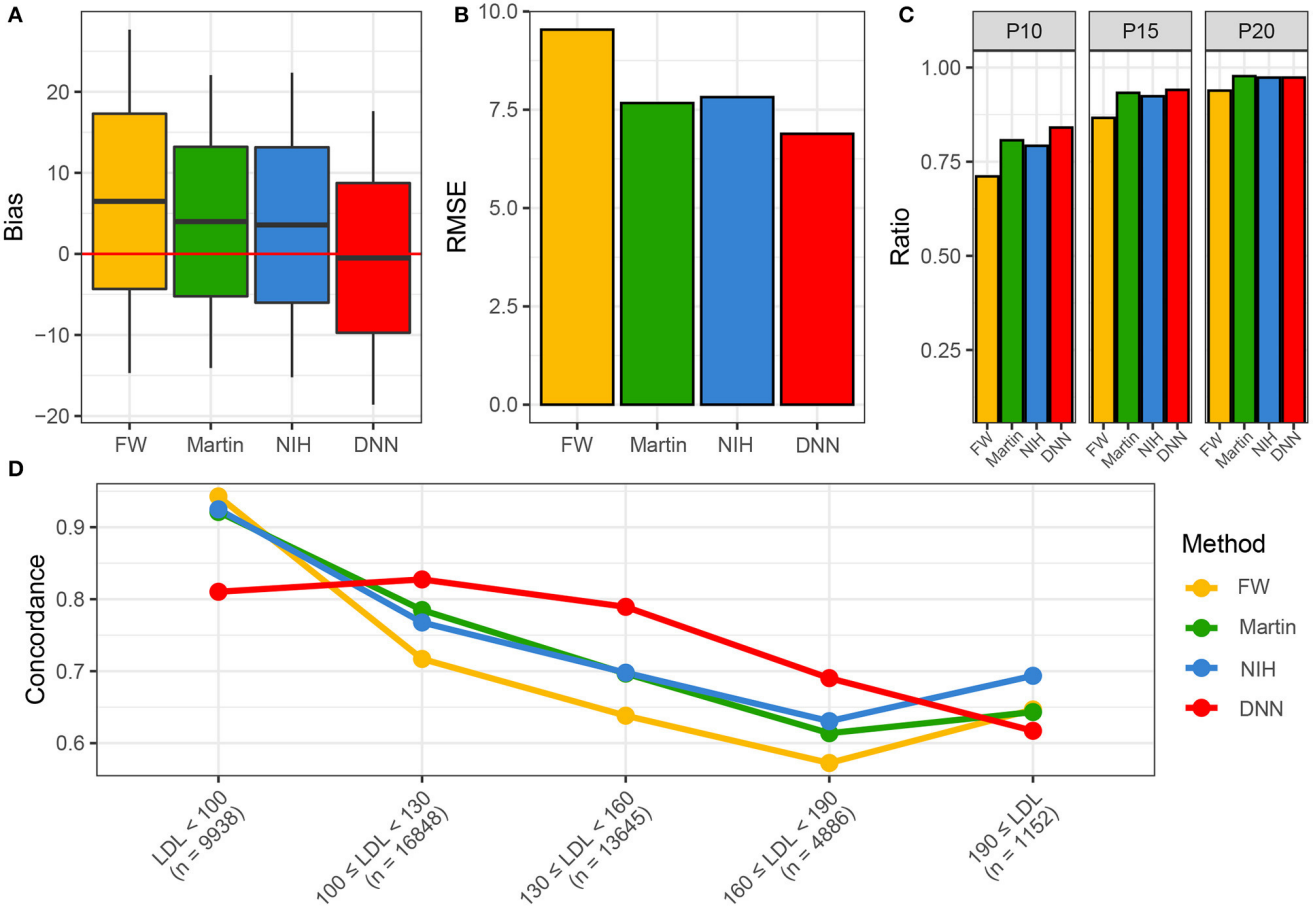
Performances of four LDL estimation methods. **(A)** Bias of four LDL estimation models. **(B)** RMSE of four LDL estimation model. **(C)** P10, 15, and 20 of four LDL estimation models. **(D)** Concordances of four LDL estimation models. DNN, deep neural network; FW, Friedewald LDL-C; LDL, Low-density lipoprotein cholesterol; NIH, NIH equation for LDL-C; Novel, novel equation for LDL-C.

Figure 5 shows the performance of various LDL equations in the external validation set. The LDL-*C*_*DNN*_ was biased the lowest from the zero (mean: 0.11 and *t*-value: 2.0 in the internal validation set and mean: −0.08 and *t*-value: 1.9 in the external validation set). The LDL-*C*_*Friedewadl*_ was biased the highest for zero (mean: 6.38 and *t*-value: 88.3 in the internal validation set and mean: 6.49 and *t*-value: 129.3 in the external validation set). The LDL-*C*_*DNN*_ had the lowest root mean-square error (RMSE), and the Friedewald LDL-C equation had the highest RMSE in both validation sets. In the external validation set, LDL-*C*_*DNN*_ had superior performance in the P10 and P15. The LDL-*C*_*DNN*_ had the highest concordance in the LDL-C range from 100 to 190 mg/dl.

**Figure 6:**
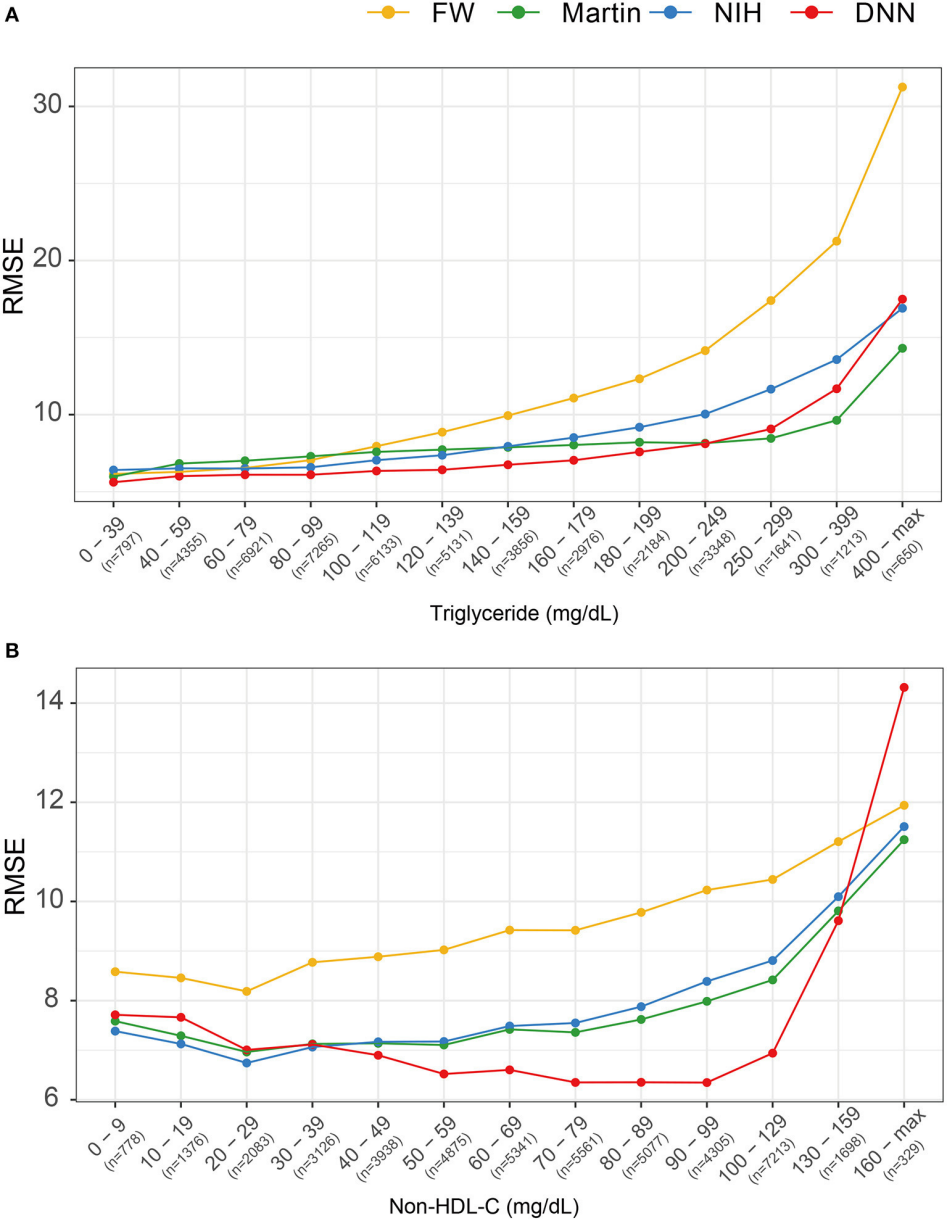
Comparison of root mean-square errors (RMSEs) for four LDL estimation models. **(A)** RMSEs for four LDL estimation models according to triglyceride classification. **(B)** RMSE for the four LDL estimation models according to non-HDL cholesterol classification.

Figure 6 describes the RMSEs of various LDL equations using the external validation set. The LDL-*C*_*DNN*_ had lowest RMSE within a TG range as high as 250 mg/dl. When analyzed by non-HDL-C ranges, LDL-*C*_*DNN*_ had the lowest RMSE values among all estimations, particularly at the lower range (40–159 mg/dl non-HDL-C range). This range has a similar meaning to 70–190 mg/dl LDL-C.

**Figure 7:**
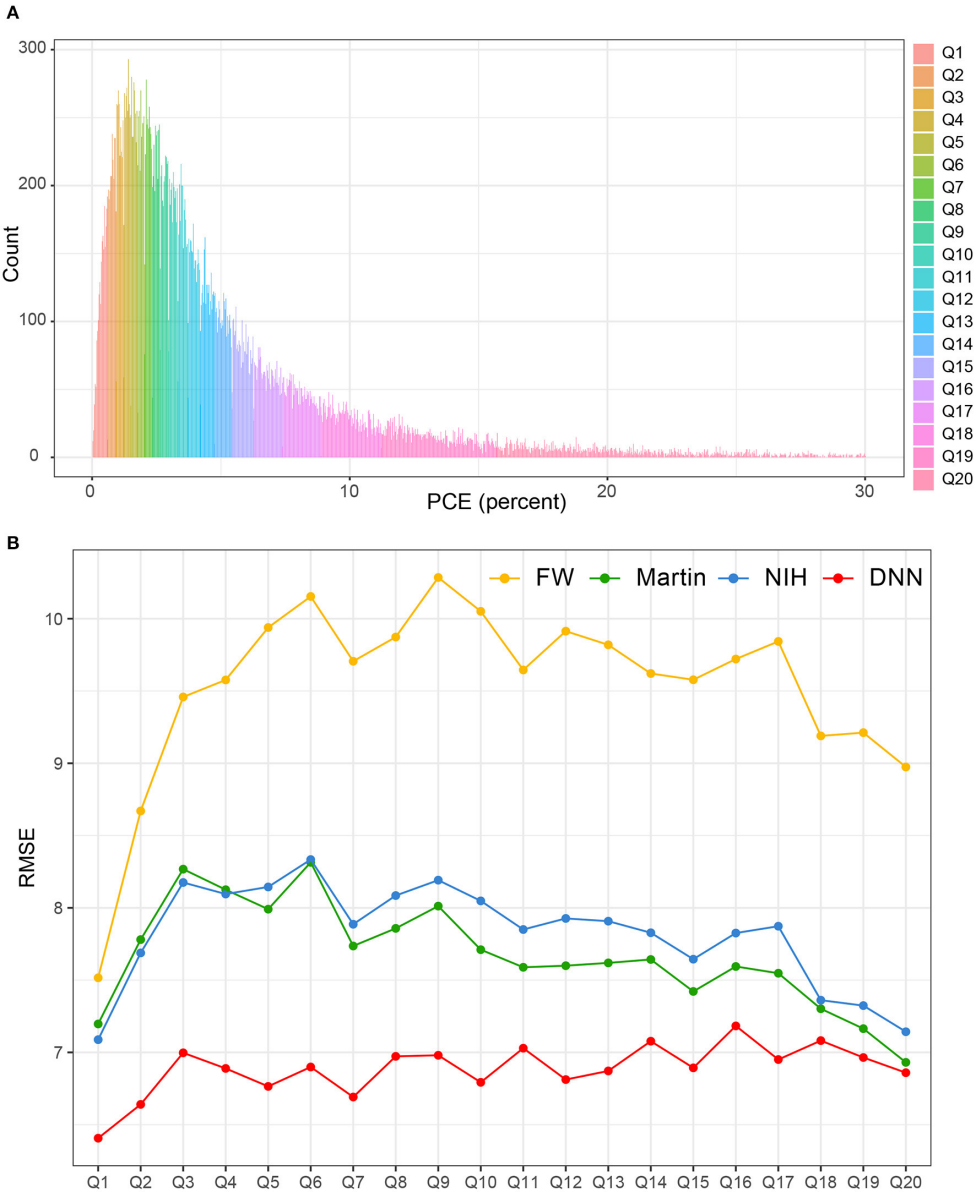
Comparison of RMSE in the four LDL estimation models across the PCE categories. PCE, pooled cohort equations. **(A)** PCE scores were classified based on the 20th decile. **(B)** The RMSEs of each LDL-C estimation methods were separately calculated according to each of the 20 PCE categories.

Figure 7A shows the distribution of PCE categories. The RMSEs of each LDL-C estimation methods were presented according to each of the 20 PCE categories (Figure 7B). The LDL-*C*_*DNN*_ had the lowest RMSE in most of the PCE score range in the external validation set.

## Discussion

In the current study, we developed the DNN method for estimating LDL. This method was a better estimator over the previous equations including Friedewald's, Martin's, and NIH formulas, showing a high agreement with LDL-C measured by a homogenous assay. In particular, the DNN method is more concordant with serum LDL-C throughout all PCE strata, meaning that RMSE is consistently low not only in low-risk CVD groups but also high-risk CVD groups.

Previous epidemiological studies have consistently revealed significant associations between blood cholesterol levels and coronary artery diseases (26). Gofman et al. (27) reported that LDL and VLDL are associated with coronary artery diseases. LDL particles are the major group for the transport of cholesterol by the LDL receptor and plasma LDL concentrations (28). Findings from familial hypercholesterolemia, a mutation in the LDL receptor gene, suggests that exposure to excessive LDL-C at an early age results in premature ASCVD (28, 29). Results from Mendelian randomization studies have indicated that polymorphisms associated with lower LDL level are associated with a lower risk of ASCVD (29, 30). These findings provide powerful evidence that LDL is an important causal factor for ASCVD risk. Therefore, several studies have tried to develop more accurate LDL-C estimation that could be used in routine clinical practice (9–11).

Since the 1970s, the Friedewald formula has been used to calculate LDL-C levels using the standard TC, HDL-C, and TG lipid profiles (9). This method assumes a fixed factor (5:1) for the TG to VLDL-C ratio. However, LDL-C estimation with the Friedewald formula underestimates LDL-C compared with ultracentrifugation or methods of direct LDL-C measurement (9). The limitations of the Friedewald formula are that it is not applicable in non-fasting subjects or those with TG levels of 400 mg/dl or more or those with LDL levels lower than 70 mg/dl (31). Furthermore, this formula could be inaccurate in patients with diabetes, kidney diseases, or liver disease, all of which have been defined as risk-enhancing factors in the ACC/AHA guidelines (32–34). These issues have become more important as cholesterol treatment has evolved to consider much lower LDL-C levels for prevention of CVD in high-risk patients.

Martin et al. developed a novel method for LDL-C estimation by applying an adjustable factor for the TG to VLDL ratio based on each individual's non-HDL-C and TG levels (10). This Martin method provided a more accurate estimate in cases of LDL-C levels lower than 70 mg/dl, as well as high TG levels (up to 400 mg/dl). This study used the VAP method for LDL-C measurement as a reference method. The Martins method still has one significant weakness: it lacks accuracy for estimating LDL-C with TG levels of more than 400 mg/dl.

Sampson et al. suggested a new equation for LDL-C estimation using data from the NIH Clinical Center (11). In their study, they used multiple least squares regression to develop an equation for VLDL-C and used multiple external validation sets including both β-quantification LDL-C and direct LDL-C tests (Roche dLDL-C and Beckman dLDL-C). The strength of the NIH method was the improved accuracy for estimating the LDL-C in individuals with low LDL-C and high TG levels. However, this study included a population with a high incidence of hypertriglyceridemia. The median TG level was 149 mg/dL (IQR, 98–253 mg/dL), and 14% of the samples had a TG level of 400 mg/dL or more. These values were relatively higher than the results of our study.

Recently, an increasing number of machine learning algorithms have been developed for predicting cardiovascular risk (35). Machine learning provides an improved performance of modeling and outcome prediction in cardiovascular medicine. Several studies have developed machine learning methods to better estimate LDL-C levels (14–16). Lee et al. (14) developed a DNN model for estimating LDL-C including 180 perceptrons, which was motivated by the novel method from the standard lipid profile (TC, HDL-C, and TG). Singh et al. (16) proposed a machine learning method utilizing random forests for LDL-C estimation using a direct LDL-C as a reference value. Tsigalou et al. (15) suggested a machine learning model to estimate LDL-C using shallow and deep machine learning methods. Although these attempts improved the accuracy for LDL-C estimation, these studies were conducted with relatively small sample sizes, and comparative analyses of the performances of machine learning and other LDL equations are needed.

In this study, we developed the DNN model for LDL estimation using the pyramid structure and selected the best DNN model using the tournament method. Then, we compared the performance of LDL-*C*_*DNN*_ to that of other recently developed LDL estimations with large multicenter data.

We used direct measurement LDL-C as a reference value. The direct LDL method using homogenous reagents improved imprecision over the previous methods and can be used more easily in the clinical setting. To date, several reagents have been developed by various manufacturers (36). Furthermore, the Centers for Disease Control and Prevention performed a manufacturer certification program with the aid of the Cholesterol Reference Method Laboratory Network to ensure global standardization and harmonization of lipid laboratory tests, which satisfy the requirement of the National Cholesterol Education Program (37).

In our results, the novel and NIH LDL-C equations had more accurate performance than the Friedewald LDL-C equation; these results were consistently concordant with those of previous studies. LDL-*C*_*DNN*_ had the lowest bias and RMSE of the four methods tested. Particularly, the higher ratio value of LDL-*C*_*DNN*_ from P20 to P10 indicates that LDL-*C*_*DNN*_ better predicts serum LDL-C within a smaller margin of error.

The concordance of LDL-*C*_*DNN*_ was superior within the LDL-C range of 100–190 mg/dl, which includes the LDL-C IQR (103–145 mg/dl) in the external validation dataset. Additionally, LDL-*C*_*DNN*_ showed superior performance in non-HDL-C ranges of 40–159 mg/dl. The range between 40 and 159 mg/dl non-HDL-C corresponds to 70–190 mg/dl LDL-C. Non-HDL-C is not unaffected by issues related to the lipoprotein specificity of serum LDL-C methods toward various ApoB-containing lipoproteins (38). Therefore, non-HDL-C is known to have better concordance with CVD risk score classification in both healthy individuals and those with hypertriglyceridemia (38).

However, we could not overcome the inaccuracy of LDL estimation for low LDL and high TG levels, which were similar with disadvantages of the existing LDL measurement formulas. This result might have been due to the relatively small number of people with low LDL and high TG because we used the data from a generally healthy population.

To overcome these shortcomings, we compared the RMSEs according to cardiovascular risk stratification using the PCE. This comparison revealed that LDL-*C*_*DNN*_ predicts LDL-C well across the entire PCE range regardless of CVD risk. PCEs were first introduced in 2013 as sex- and race-specific tools for estimating ASCVD risk (23). PCEs included not only age, sex, and race but also established cardiovascular risk factors such as smoking status, systolic blood pressure, hypertension treatment status, diabetes status, and total and HDL-C levels. PCEs were considered in the context of a particular patient's circumstances when deciding whether to use statin therapy (39). LDL-*C*_*DNN*_ consistently predicted LDL-C well in participants with low or high CVD risk. Our findings suggest that the DNN method could allow for risk-stratified care management and reduce ASCVD risk by achievement of LDL-C targets regardless of risk levels.

This study has several limitations. First, we used a reference value based on the direct homogenous assay of LDL-C instead of the β-quantification (BQ) method, which is considered the gold standard for LDL-C measurement. Therefore, the results of the current study should be interpreted with caution. Comparison of LDL-*C*_*DNN*_ and BQ method is needed in further studies. The BQ procedure, which relies on preparative ultracentrifugation has been the established reference measurement procedure for HDL-C and LDL-C (6); however, this method is a highly manual technique requiring significant laboratory skill and expense, which is not suitable in the clinical setting (11). Homogenous automated methods for direct measurement of LDL-C are well-suited to routine clinical application and have an assay precision generally within the level stated in NCEP guidelines (40). Therefore, the 2019 EAS/ESC guidelines suggested that both homogenous enzymatic methods and ultracentrifugation for LDL-C measurement are useful for clinicians (3). Considering the real-world data in Korea, the utilization of homogenous assays has practical merits. Two recent studies, which developed machine learning method for the estimation LDL-C, also used direct homogenous assay of LDL-C (15, 16).

Second, factors related to abnormal lipoprotein composition (e.g., diabetes, obesity, kidney diseases, and liver diseases) were not available for this analysis like other LDL equations. Third, LDL-*C*_*DNN*_ was more concordant when TG levels were 400 mg/dl or less in the external validation set, which is similar with other LDL equations such as the Friedewald and novel methods. Fourth, since our data only included Korean subjects, there is a limitation in applying our result to other ethnic groups. Additional validation sets are needed, including other race/ethnic groups and subgroups. Fifth, although DNN is still useful for application in predictive models of large-scale studies, it is also important to consider how to link it to practical use. Despite of theses weakness, our study used various Korean population datasets obtained by well-validated laboratories. To the best of our knowledge, this is the first study to compare the performance of the DNN method with that of other LDL estimation methods using a large sample and multicenter, real-world dataset in an East Asian population. Second, we selected the DNN model with the best performance using a model selection approach that consisted of testing all possible combinations.

## Conclusion

The DNN method offers a more precise LDL estimation in all PCE strata and may be particularly helpful in managing patients' cholesterol levels based on their ASCVD risk. More studies are needed to determine how the DNN method can better predict LDL-C within low LDL and high TG ranges. Additionally, longitudinal studies are needed to predict CVD mortality and morbidity using the DNN method.

## Data Availability Statement

The raw data supporting the conclusions of this article will be made available by authors' permission.

## Ethics Statement

The studies involving human participants were reviewed and approved by this study was approved by the Institutional Review Board of Severance Hospital (IRB No. 4-2020-0323). The patients/participants provided their written informed consent to participate in this study.

## Author Contributions

Y-JK, HL, J-WL, and H-JC contributed to the conception or design of the work and contributed to the acquisition, analysis, or interpretation of the data and drafted the manuscript. All authors critically revised the manuscript, provided final approval, and agree to be accountable for all aspects of the work, ensuring integrity, and accuracy.

## Funding

This work was supported by the Technology Innovation Program (20002781, A Platform for Prediction and Management of Health Risk Based on Personal Big Data and Lifelogging) funded by the Ministry of Trade, Industry and Energy, Korea, to J-WL; Korea Institute of Planning and Evaluation for Technology in Food, Agriculture and Forestry (IPET) through High Value-added Food Technology Development Program funded by Ministry of Agriculture, Food and Rural Affairs (MAFRA; 321030051HD030) to J-WL and Y-JK; the Institute for Information & Communications Technology Promotion grant funded by the Korea government (MSIT; 2019-31-1293, Autonomous digital companion framework and application) to H-JC; and the National Research Foundation of Korea grant funded by the Korea government (MEST; NRF-2019R1A2C1010043) to HL.

## Conflict of Interest

The authors declare that the research was conducted in the absence of any commercial or financial relationships that could be construed as a potential conflict of interest.

## Publisher's Note

All claims expressed in this article are solely those of the authors and do not necessarily represent those of their affiliated organizations, or those of the publisher, the editors and the reviewers. Any product that may be evaluated in this article, or claim that may be made by its manufacturer, is not guaranteed or endorsed by the publisher.
