# Supplementary material for: Comparison of a Machine Learning Method and Various Equations for Estimating Low-Density Lipoprotein Cholesterol in Korean Populations
Source: Front Cardiovasc Med. 2022 Feb 10;9:824574. doi: 10.3389/fcvm.2022.824574 (PMC8866707; doi:10.3389/fcvm.2022.824574)
Supplement: Supplementary file 1 [file Table_1.DOCX]

**Supplementary Table 1. Baseline characteristics of the study populations among the three datasets**

| Variables | Derivation dataset (N = 91,002) | | Internal dataset (N = 38,928) | | External dataset (N = 46,469) | |
| --- | --- | --- | --- | --- | --- | --- |
|  | Mean ± SD or (min, max) | Median (IQR) | Mean ± SD | Median (IQR) | Mean ± SD | Median (IQR) |
| Age | 48.6 ± 11.5 | 49 (41–56) | 48.5 ± 11.4 | 49 (41–56) | 54 ± 8.9 | 53 (48–59) |
| Sex (male), n (%) | 48667 (53.5) | 48667 (53.5) | 20880 (53.6) | 20880 (53.6) | 35387 (76.2) | 35387 (76.2) |
| SBP, mmHg | 121.6 ± 14.4 | 121 (111–131) | 121.6 ± 14.4 | 121 (111–131) | 120.6 ± 15.4 | 119 (110–131) |
| TC, mg/dl* | 198.3 (77, 696) | 196 (173–222) | 198.3 (77.466) | 196 (173–221) | 197.1 (73, 450) | 196 (173–219) |
| HDL-C, mg/dl* | 54.4 (9, 201) | 53 (45–62) | 54.4 (9, 133) | 53 (45–62) | 52.3 (13, 162) | 50 (43–60) |
| Triglyceride, mg/dl* | 127.3 (16, 3271) | 105 (75–153) | 127.7 (8, 1670) | 105 (75–153) | 133.3 (16, 1798) | 113 (79–163) |
| LDL-D, mg/dl* | 124.8 (10, 365) | 123 (102–145) | 124.8 (18, 386) | 123 (102–146) | 124.6 (13, 356) | 124 (103–145) |
| LDL$C_{Friedwald}$, mg/dl* | 118.4 (-214.2, 360.2) | 116.8 (95.6–139.6) | 118.4 (-75.4, 370.2) | 116.6 (95.6–139.4) | 118.1 (-122.6, 370.4) | 117 (96–138.8) |
| LDL-$C_{Martin}$,, mg/dl* | 120.4 (-89.3, 362.7) | 118.5 (98–140.9) | 120.4 (9.7, 367) | 118.4 (97.8–140.7) | 120.6 (-14.1, 368) | 119.4 (99.4–140.2) |
| LDL-$C_{NIH}$, mg/dl* | 121.1 (0.4, 354.2) | 119.4 (98.2–142.2) | 121.1 (7.5, 374.3) | 119.2 (98–142) | 121 (3.9, 373.7) | 119.8 (99–141.5) |
| Hypertension, n (%) | 8269 (9.1) | 8269 (9.1) | 3558 (9.1) | 3558 (9.1) | 10422 (22.4) | 10422 (22.4) |
| Diabetes, n (%) | 4073 (4.5) | 4073 (4.5) | 1732 (4.4) | 1732 (4.4) | 4491 (9.7) | 4491 (9.7) |
| Smoking, n (%) | 15031 (16.5) | 15031 (16.5) | 6428 (16.5) | 6428 (16.5) | 9712 (20.9) | 9712 (20.9) |
| PCE (high risk), n (%) | 10188 (11.2) | 10188 (11.2) | 4251 (10.9) | 4251 (10.9) | 9109 (19.6) | 9109 (19.6) |

HDL, high-density lipoprotein; IQR, internal quartile range; LDL, low-density lipoprotein; PCE, pooled cohort equation; SD, standard deviation; SBP, systolic blood pressure; TC, Total cholesterol;

*Data for lipid profiles are represented as mean (min, max)
